# A novel nomogram model to predict the overall survival of patients with retroperitoneal leiomyosarcoma: a large cohort retrospective study

**DOI:** 10.1038/s41598-022-16055-z

**Published:** 2022-07-13

**Authors:** Chao Huang, Qiu-Ping Yu, Hao Li, Zichuan Ding, Zongke Zhou, Xiaojun Shi

**Affiliations:** 1 Department of Orthopaedics, West China Hospital of Sichuan University, No. 37 Guoxue Alley, Wuhou District, Chengdu, 610041 Sichuan People’s Republic of China; 2 Health Management Center, West China Hospital of Sichuan University, No. 37 Guoxue Alley, Wuhou District, Chengdu, 610041 Sichuan People’s Republic of China

**Keywords:** Cancer, Cancer prevention, Cancer therapy, Sarcoma

## Abstract

Retroperitoneal leiomyosarcomas (RLS) are the second most common type of retroperitoneal sarcoma and one of the most aggressive tumours. The lack of early warning signs and delay in regular checkups lead to a poor prognosis. This study aims to create a nomogram to predict RLS patients' overall survival (OS). Patients diagnosed with RLS in the Surveillance, Epidemiology, and End Results (SEER) database between 2000 and 2018 were enrolled in this study. First, univariable and multivariable Cox regression analyses were used to identify independent prognostic factors, followed by constructing a nomogram to predict patients' OS at 1, 3, and 5 years. Secondly, the nomogram's distinguishability and prediction accuracy were assessed using receiver operating characteristic (ROC) and calibration curves. Finally, the decision curve analysis (DCA) investigated the nomogram's clinical utility. The study included 305 RLS patients, and they were divided into two groups at random: a training set (216) and a validation set (89). The training set's multivariable Cox regression analysis revealed that surgery, tumour size, tumour grade, and tumour stage were independent prognostic factors. ROC curves demonstrated that the nomogram had a high degree of distinguishability. In the training set, area under the curve (AUC) values for 1, 3, and 5 years were 0.800, 0.806, and 0.788, respectively, while in the validation set, AUC values for 1, 3, and 5 years were 0.738, 0.780, and 0.832, respectively. As evidenced by the calibration curve, the nomogram had high prediction accuracy. Moreover, DCA revealed that the nomogram had high clinical utility. Furthermore, the risk stratification system based on the nomogram could effectively categorise patients into three mortality risk subgroups. Therefore, the developed nomogram and risk stratification system may aid in optimising the treatment decisions of RLS patients to improve treatment prognosis and maximise their healthcare outcomes.

## Introduction

Retroperitoneal tumours are exceptionally rare, accounting for only 0.1–0.2% of malignant tumours^1,2^. Soft tissue sarcomas are the most common type, with 70–80% of retroperitoneal soft tissue tumours being malignant. Rretroperitoneal leiomyosarcoma (RLS) is the second most prevalent retroperitoneal soft tissue sarcoma after liposarcomas^3,4^. There are no specific signs or symptoms associated with RLS, and early detection is difficult due to the large and deep potential retroperitoneal space. Furthermore, most diagnoses are made when the tumour infiltrates and squeezes the surrounding organs, resulting in clinical symptoms. RLS can be diagnosed by core biopsy and computed tomography^5^. While complete resection remains the gold standard of treatment, with a 50% chance of a 5 years survival rate^6^, adjacent organs are always infiltrated by tumours and are typically removed concurrently. The invasion of prominent blood vessels by RLS is a common cause of the incomplete removal of a tumour, resulting in a recurrence rate of up to 80% after resection^7^. RLS also exhibits malignant characteristics, resulting in a poor prognosis and a low 5-year survival rate^8^. Therefore, reliable prognostic factors are required to accurately predict RLS patients' prognoses.

Several studies have concluded that the maximum tumour diameter, tumour differentiation degree, whether the operation was radical, and the amount of intra-operative blood loss are critical factors affecting the prognosis of primary RLS^8–12^. The effectiveness of postoperative adjuvant radiotherapy and chemotherapy is debatable^13,14^. The prognosis of a disease cannot be accurately predicted by relying solely on a single indicator, and satisfactory results are challenging to achieve. The use of multiple independent indicators could improve prediction credibility and efficiency. Although numerous risk factors for mortality associated with RLS have been identified, there is no universally accepted scoring system available for predicting patients’ mortality. Given the various clinic-pathological variables that can affect a patient's prognosis, a tool that integrates critical prognostic indicators is urgently required to aid in therapy and improve patients’ life quality.

The nomogram is now widely accepted as a simple multivariable oncology visualisation tool for predicting and quantifying individual survival outcomes^15,16^. Compared to tumour-node-metastasis (TNM) staging classification, nomogram can more accurately estimate an individual's overall survival (OS) by combining and integrating critical variables to aid in clinical decision-making and the development of precision medicine^17,18^. However, no nomogram for RLS patients has been available to our knowledge. Therefore, this study aims to identify independent prognostic factors for RLS patients by analysing relevant data from the Surveillance, Epidemiology, and End Results (SEER) database, as well as to develop a new nomogram and risk stratification system to predict RLS patients' 1, 3, and 5 years OS.

## Methods

### Database

The SEER database is the largest publicly available cancer dataset. It is a population-based cancer registry that spans multiple geographic regions and covers approximately 30% of the US population, and includes cancer incidence and prevalence statistics and public demographic information segmented by age, gender, race/ethnic origin, year of diagnosis, marital status, surgery, radiotherapy, chemotherapy, and TNM stage^19^. Informed consent from patients and ethics committee approval was not required in this study because the content excluded human subjects or individual privacy.

### Patient selection

**Figure 1:**
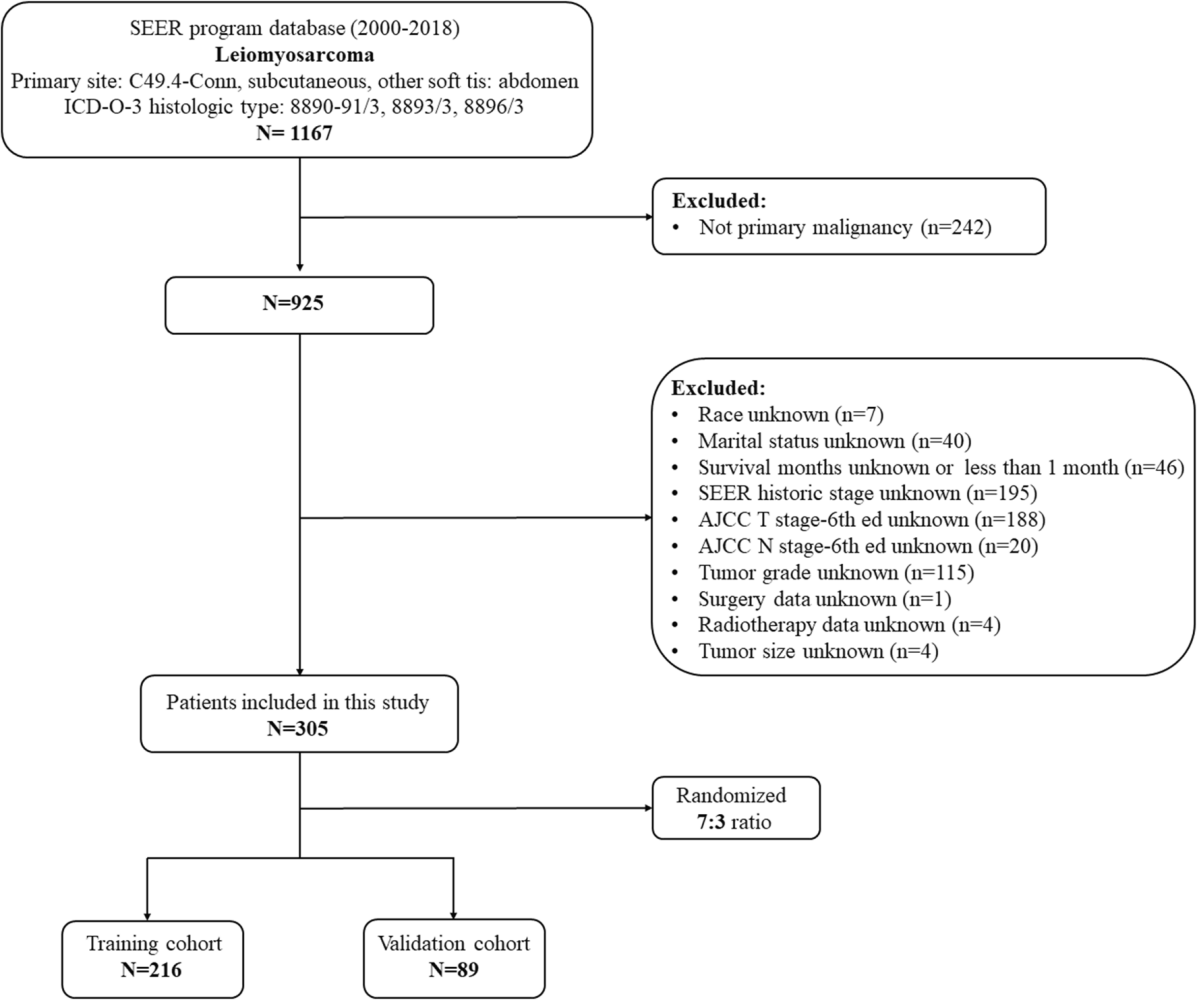
The flowchart of patient selection.

RLS patients from 2000 to 2018 were determined using SEER Stat 8.3.9.2. Inclusion criteria for the study were as follows: (1) RLS was the histological type; (2) primary tumor; (3) complete T and N staging information; and (4) complete follow-up data. While the following were the exclusion criteria: (1) lack of crucial detailed information, such as age, tumour size, tumour grade, TNM stage, histological type, surgery, survival time, and cause of death; (2) survival time < 1 month. Patients who met the above criteria were randomly assigned to one of two groups: a training set (70%) and a validation set (30%), and the classification was done with R software (Fig. 1). We constructed a nomogram based on the training set and evaluated it against the validation set.

### Variable definitions

Patients' demographics (age, gender, race, and marital status), as well as their disease characteristics (tumour size, tomour stage, and tumour grade), and treatment methods (surgery, chemotherapy, and radiotherapy), were all included in this study. The variables of age and gender were converted into categorical variables. There were three categories of people: blacks, whites, and others. For each of the following: marriage status, radiotherapy, chemotherapy, and surgery, a yes or no response was given. The tumour stages were divided into three categories: local, regional, and distant^20^; the tumour grades were divided into grades I/II and III/IV. The AJCC T stage was split into T1 and T2, and the AJCC N stage was divided into N0 and N1. The time interval between the initial diagnosis and the date of death from any cause was defined as OS, which was this study's endpoint.

### Statistical analysis

The appropriate tumour size cut-off value was determined using X-tile software. Univariable and multivariable Cox regression analyses were used to investigate independent prognostic factors of OS, then a nomogram to predict 1, 3, and 5 years OS was built. A calibration curve was generated to compare the nomogram's predicted probability with the observed results. A decision curve analysis (DCA) was used to demonstrate the nomogram’s clinical utility. Furthermore, total scores for all patients in the training set were estimated, a time-dependent receiver operating characteristic (ROC) curve for the nomogram was established, and the area under the curve (AUC) was calculated to show the nomogram's discrimination for 1, 3, and 5 years OS. The optimal cut-off value for the total score was determined using the x-tile software, and a risk stratification system was established to accurately stratify all patients' mortality risks. The survival function and variables were estimated and compared using Kaplan–Meier estimates and the log-rank test. Chi-square and student t-tests were used to compare categorical and continuous variables, respectively. All statistical analyses in this study were conducted using SPSS (version 22.0), X-tile (version 3.6.1), and R software (version 4.0.3, https://www.r-project.org/), with a p-value of less than 0.05 considered statistically significant. All methods were carried out in accordance with relevant guidelines and regulations.

### Ethical approval and consent to participate

All methods were carried out in accordance with relevant guidelines and regulations. Data extraction and usage has been approved by SEER Program. All the data can be found in the SEER dataset: https://seer.cancer.gov/seerstat/. We obtained access to the SEER database after obtaining permission to access research data files with the reference number 16336-Nov2020.

## Results

### Baseline characteristics

We found 305 patients with RLS who met our criteria in the SEER database (Fig. 1). They were randomly divided into a training set (n = 216) and a validation set (n = 89) in a 7:3 ratio. Age was converted into two categorical variables, < 60 and ≥ 60 years old^21^. The best cut-off values for tumour size were 76 and 132 mm. Therefore, tumour size was categorized into three variables: < 76, 76–132, and > 132 mm (Supplementary File 1). RLS patients' baseline demographics and clinical characteristics are listed in Table 1.

**Table 1:** The baseline demographics and clinical characteristics of patients with retroperitoneal leiomyosarcoma in this cohort retrospective study.

Variables	Training set		Validation set		Total	
	216	70.00%	89	30.00%	305	100.00%
**Age (years old)**						
< 60	108	50.00%	36	40.45%	144	47.21%
≥ 60	108	50.00%	53	59.55%	161	52.79%
**Race**						
Black	21	9.72%	15	16.85%	36	11.80%
White	174	80.56%	69	77.53%	243	79.67%
Other	21	9.72%	5	5.62%	26	8.53%
**Sex**						
Female	147	68.06%	54	60.67%	201	65.90%
Male	69	31.94%	35	39.33%	104	34.10%
**Tumor grade**						
Grade I/II	80	37.04%	35	39.33%	115	37.70%
Grade III/IV	136	62.96%	54	60.67%	190	62.30%
**Tumor size (mm)**						
< 76	73	33.80%	27	30.34%	100	32.79%
76–132	61	28.24%	51	57.30%	112	36.72%
> 132	82	37.96%	11	12.36%	93	30.49%
**AJCC T stage**						
T1	34	15.74%	18	20.22%	52	17.05%
T2	182	84.26%	71	79.78%	253	82.95%
**AJCC N stage**						
N0	210	97.22%	85	95.51%	295	96.72%
N1	6	2.78%	4	4.49%	10	3.28%
**Tumor stage**						
Localized	116	53.70%	37	41.57%	153	50.16%
Regional	62	28.70%	34	38.20%	96	31.48%
Distant	38	17.60%	18	20.23%	56	18.36%
**Surgery**						
No	25	11.57%	18	20.22%	43	14.10%
Yes	191	88.43%	71	79.78%	262	85.90%
**Radiotherapy**						
No	136	62.96%	59	66.29%	195	63.93%
Yes	80	37.04%	30	33.71%	110	36.07%
**Chemotherapy**						
No	112	51.85%	68	76.40%	180	59.02%
Yes	104	48.15%	21	23.60%	125	40.98%
**Marital status**						
No	85	39.35%	29	32.58%	114	37.38%
Yes	131	60.65%	60	67.42%	191	62.62%

### Identification of independent prognostic factors

The following variables were included in the univariable Cox regression analysis: age, gender, race, AJCC T stage, AJCC N stage, surgery, radiotherapy, chemotherapy, tumour size, tumour grade, tumour stage, and marital status, and results are shown in Table 2. Tumour size, tumour stage, tumour grade, AJCC T stage, surgery, chemotherapy, and radiotherapy were all OS-related factors (*p* < 0.05). These variables were then used in a multivariable Cox regression analysis to reduce confounding variables (Table 2). Finally, tumour grade, tumour size, tumour stage, and surgery were identified as independent prognostic factors of OS in RLS patients (Table 2). Tumour size (> 132 mm) (HR = 2.332, *p* < 0.001), grade III/IV (HR = 1.659, *p* < 0.05), and distant metastasis (HR = 2.647, *p* < 0.01) were all associated with a higher mortality risk than other factors.

**Table 2:** The univariable and multivariable Cox regression analyses of patients with retroperitoneal leiomyosarcoma in this cohort retrospective study.

Characteristics	Univariable analysis		Multivariable analysis	
	HR (95% CI)	P-value	HR (95% CI)	P-value
**Age (years old)**				
< 60	Reference			
≥ 60	1.341 (0.963–1.867)	0.082		
**Race**				
Black	Reference			
White	1.257 (0.694–2.279)	0.451		
Other	1.450 (0.669–3.141)	0.347		
**Sex**				
Male	Reference			
Female	0.728 (0.513–1.034)	0.076		
**Tumor grade**				
Grade I/II	Reference		Reference	
Grade III/IV	1.940 (1.338–2.814)	< 0.001	1.659 (1.131–2.434)	0.01
**Tumor size (mm)**				
< 76	Reference		Reference	
76–132	1.732 (1.098–2.732)	0.018	1.919 (1.207–3.050)	0.006
> 132	2.653 (1.765–3.988)	< 0.001	2.332 (1.528–3.558)	< 0.001
**AJCC T stage**				
T1	Reference			
T2	1.798 (1.082–2.988)	0.023		
**AJCC N stage**				
N0	Reference			
N1	2.037 (0.832–4.987)	0.119		
**Tumor stage**				
Localized	Reference		Reference	
Regional	1.592 (1.084–2.338)	0.018	1.399 (0.938–2.085)	0.099
Distant	5.368 (3.425–8.413)	< 0.001	2.647 (1.521–4.607)	0.001
**Surgery**				
No	Reference		Reference	
Yes	0.170 (0.107–0.268)	< 0.001	0.222 (0.129–0.382)	< 0.001
**Radiotherapy**				
No	Reference			
Yes	0.653 (0.447–0.953)	0.027		
**Chemotherapy**				
No	Reference			
Yes	2.095 (1.466–2.996)	< 0.001		
**Marital status**				
No	Reference			
Yes	0.959 (0.685–1.342)	0.807		

HR: hazard ratio; CI: confidence interval; AJCC: American Joint Committee on Cancer.

### Construction and validation of the prognostic nomogram

**Figure 2:**
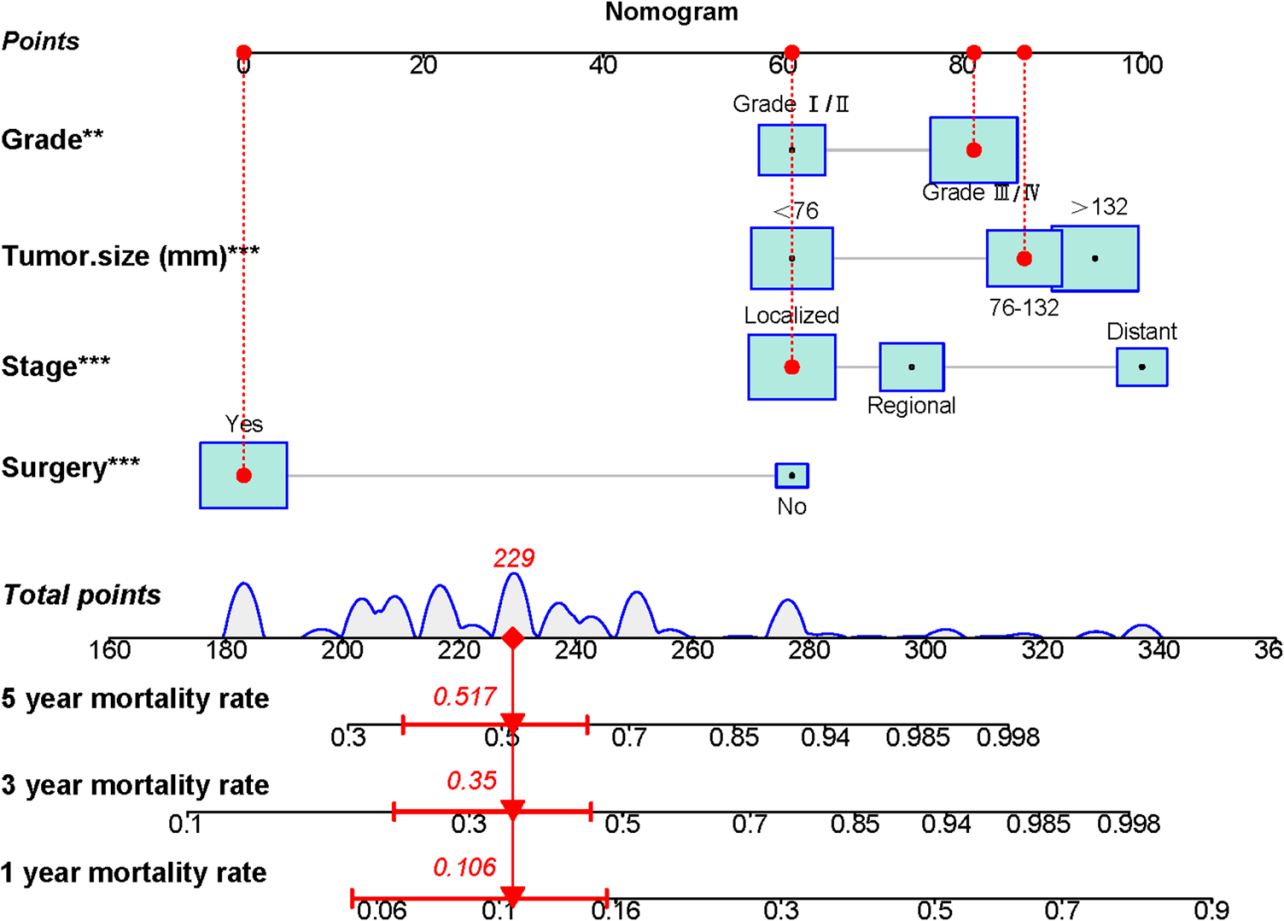
This prognostic nomogram predicts 1, 3, and 5 years OS probability in retroperitoneal leiomyosarcoma patients. Specifically, when a patient with primary retroperitoneal leiomyosarcoma comes to the clinic room for consulting his or her OS probability, we can sum each point of the above four independent prognostic factors to obtain a total point and draw a vertical line from total points row to the bottom timeline to obtain his or her mortality rate at the corresponding time. The survival probability at the corresponding time can be obtained by subtracting the mortality rate from 1. For example, a retroperitoneal leiomyosarcoma patient with a 100 mm localized metastasis and Grade III tumor and underwent surgery. The corresponding nomogram total points of this patients is 87 (100 mm) + 81 (Grade III) + 61 (Localized metastasis) + 0 (Underwent surgery) = 229, and his or her mortality rate at 1, 3, and 5 years were 10.6%, 35%, and 51.7%, respectively, while his or her corresponding OS probabilities at 1, 3, and 5 years were 89.4%, 65%, and 48.3%. Figure was generated by R software version 4.03 (http://www.r-project.org/).

**Figure 3:**
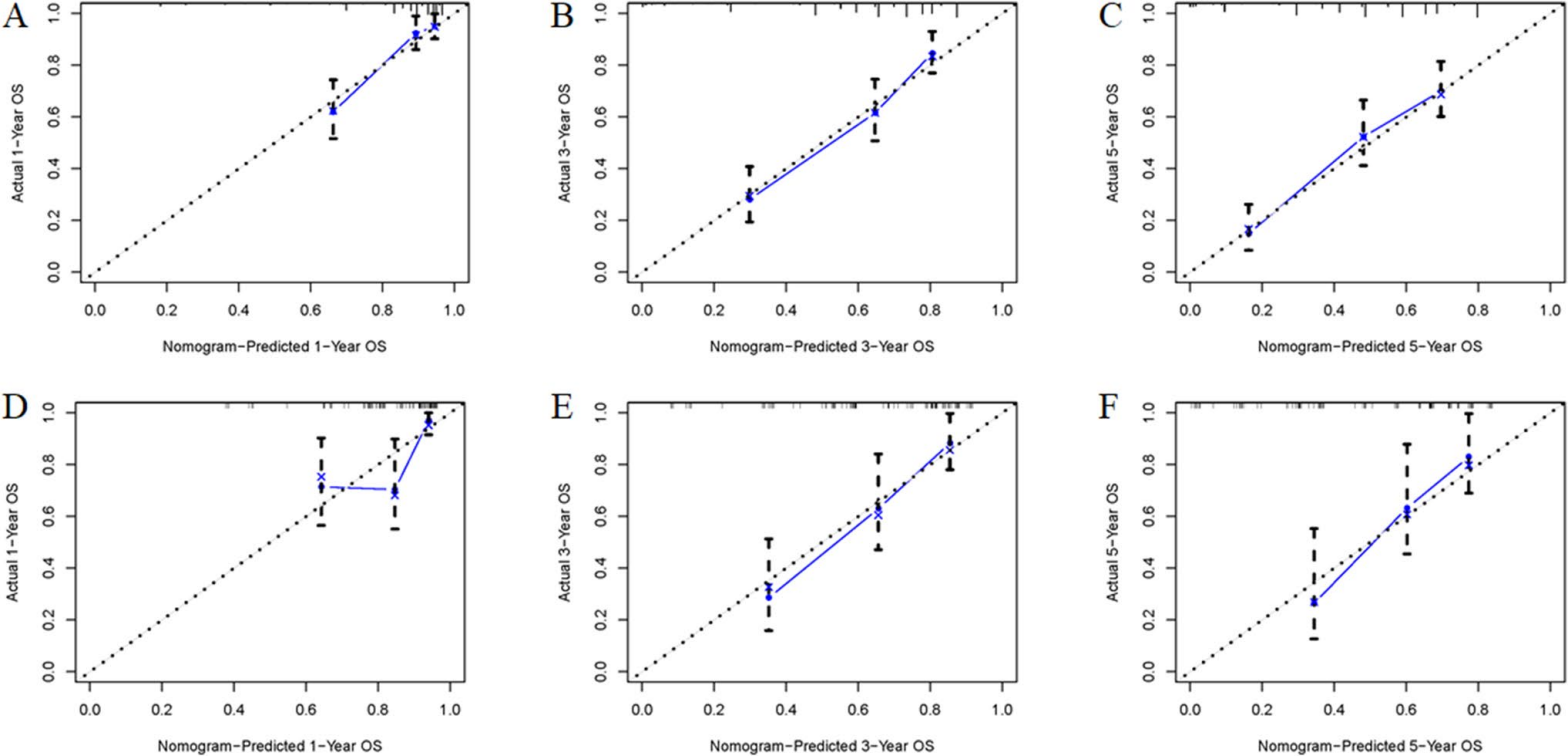
Calibration curves of the nomogram for predicting 1, 3, and 5 years OS in the training set (**A**–**C**) and 1, 3, and 5 years OS in the validation set (**D**–**F**). Figure was generated by R software version 4.03 (http://www.r-project.org/).

**Figure 4:**
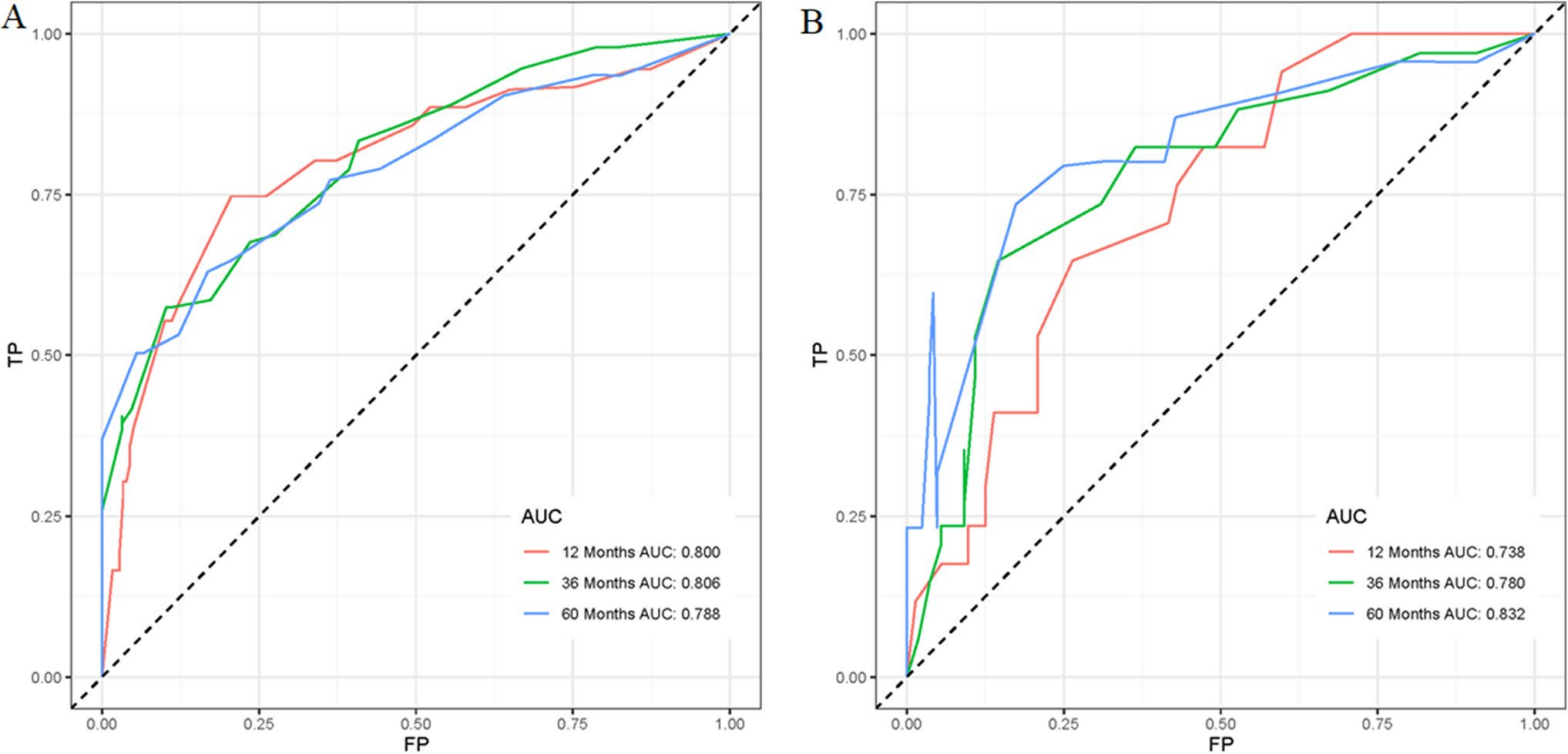
ROC curves for OS prediction of patients with retroperitoneal leiomyosarcomas. ROC curves of 1, 3, and 5 years in the training set (**A**), and ROC curves of 1, 3, and 5 years in the validation set (**B**) in this cohort retrospective study. Figure was generated by R software version 4.03 (http://www.r-project.org/).

**Figure 5:**
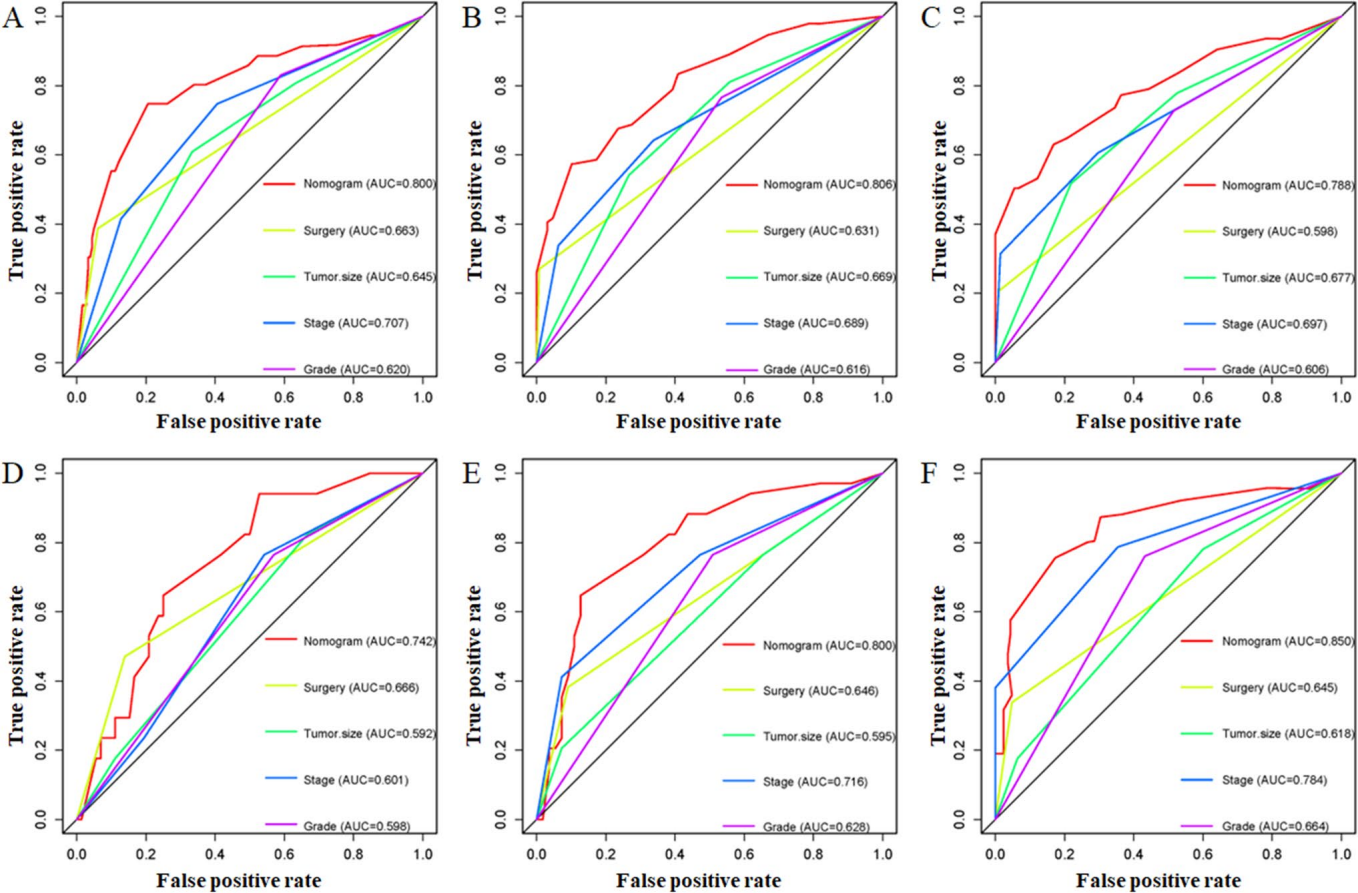
The comparison of the prediction accuracy between the nomogram and independent prognostic predictors. The ROC curves of nomogram and all independent prognostic predictors at 1 (**A**), 3 (**B**), and 5 (**C**) years in the training set and at 1 (**D**), 3 (**E**), and 5 (**F**) years in the validation set. Figure was generated by R software version 4.03 (http://www.r-project.org/).

**Figure 6:**
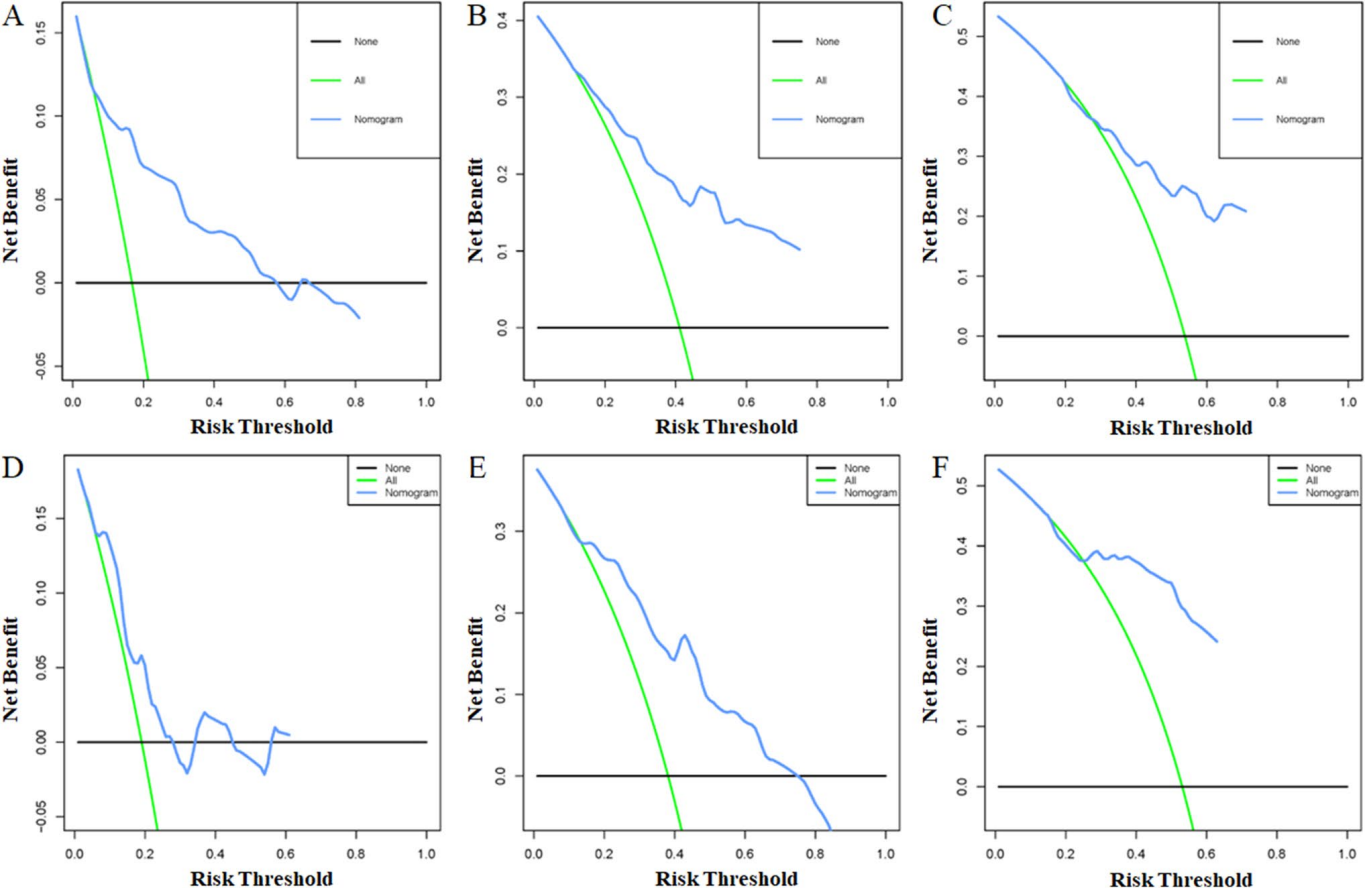
DCA of the nomogram for predicting the 1 (**A**), 3 (**B**) and 5 (**C**) year OS in the training set and the 1 (**D**), 3 (**E**) and 5 (**F**) year OS in the validation set. Figure was generated by R software version 4.03 (http://www.r-project.org/).

Based on the above-mentioned four independent prognostic factors, a nomogram was generated to predict the OS of RLS patients (Fig. 2). We can calculate each patient's survival probability by adding specific points of each independent prognostic factor to this nomogram (Supplementary File 2). Smaller tumour size, lower tumour grade, localized metastasis, and underwent surgical treatment were all protective factors for RLS patients, as shown in Fig. 2. In comparison, larger tumour size, higher tumour grade, distant metastasis, and lack of surgical treatment did not contribute to those patients' good prognosis. The calibration curves for training and validation sets demonstrated the nomogram's good differentiating abilities, the prediction results and actual observation results were highly consistent (Fig. 3). According to ROC curves, the AUCs for predicting 1, 3, and 5 years OS in the training set were 0.800, 0.806, and 0.788, respectively, whereas the AUCs for predicting 1, 3, and 5 years OS in the validation set were 0.738, 0.780, and 0.832, respectively (Fig. 4). Meanwhile, the nomogram was compared with each independent prognostic factor (Fig. 5). The nomogram exhibited significant prediction accuracy in both the training and validation sets, as depicted in Fig. 5. Furthermore, DCA revealed that the nomogram had a significant positive net income at various time points, indicating that it could be used in clinical settings (Fig. 6).

### Risk stratification system for OS

**Figure 7:**
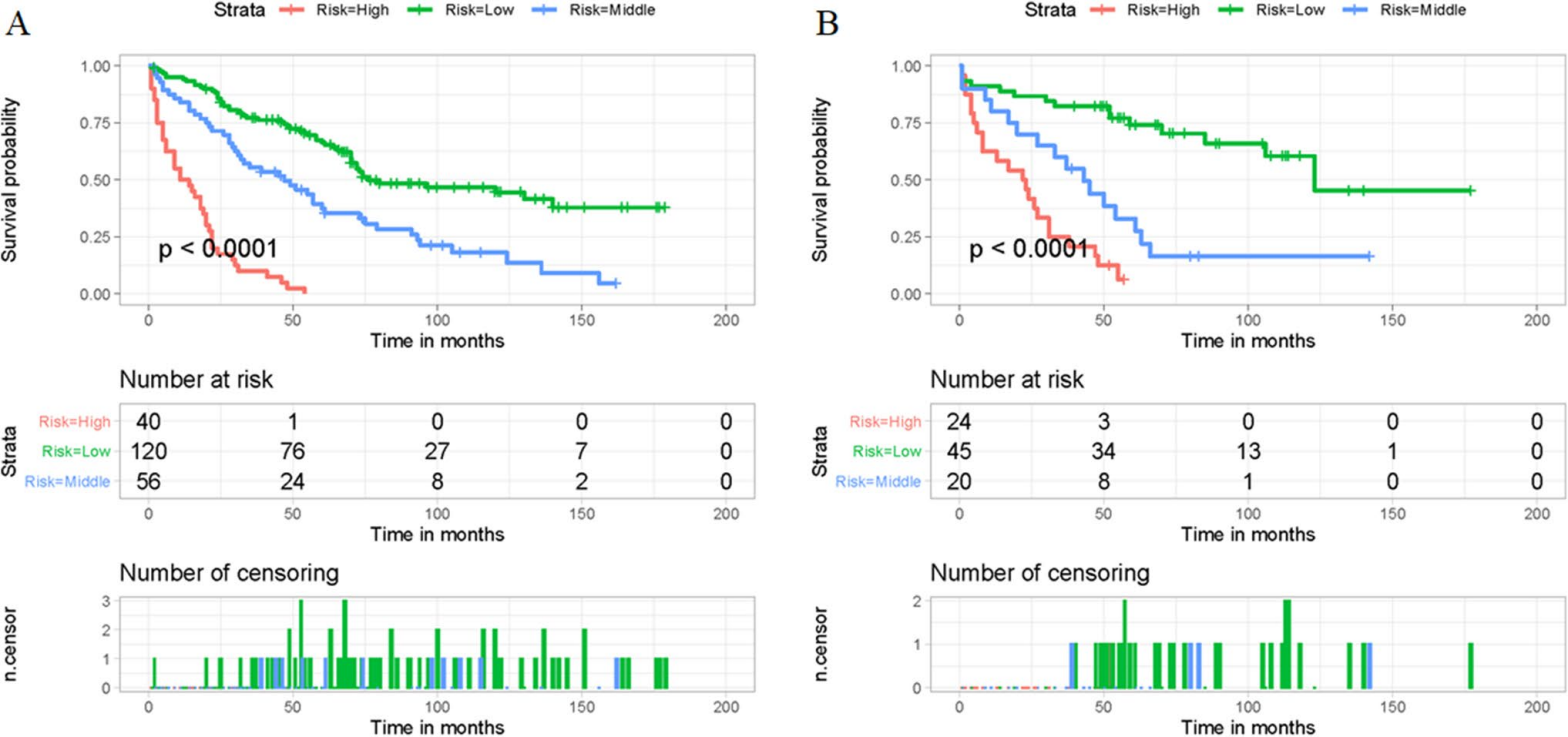
Kaplan–Meier survival analysis for the training (**A**) and validation sets (**B**) in this cohort retrospective study. Figure was generated by R software version 4.03 (http://www.r-project.org/).

Mortality risk stratification is of great significance for guiding patient management. Risk stratification and patient survival prediction could be used to test the nomogram's predictive utility further. The nomogram was used to calculate the total point of all patients in this study. The X-tile software was used to determine the best cut-off values for the total point, 230 and 256. As a result, OS total points were categorized into < 230, 230–256, and > 256 (Supplementary File 1). Those patients were then divided into three mortality risk subgroups: low (< 230), middle (230–256), and high (> 256), and Kaplan–Meier method was used to show the impact on long-term survival (Fig. 7). Kaplan–Meier survival curves of the three subgroups in the training and validation sets were all significantly different (*p* < 0.001), as shown in Fig. 7. Patients with high mortality risk had a lower OS rate than those with middle or low mortality risk, indicating that the nomogram-based risk stratification system was also predictive.

## Discussion

RLS refers to a malignant mesenchymal tissue sarcoma that originates in the smooth muscle tissue of the retroperitoneum. It is frequently found in retroperitoneal blood vessels, spermatic cords, and remnant embryonic tissues. RLS is relatively rare, accounting for approximately 20% of retroperitoneal soft tissue sarcomas, second in frequency only to liposarcoma^1,22^. Mankin et al*.* studied 1,256 patients with high-grade malignant soft tissue tumours from the FOXPRO database. They found that leiomyosarcoma accounted for less than 5% of the cases but had the highest mortality rate (up to 50%)^23^. Meanwhile, these patients died on average 3 years after diagnosed^23^. Clinical outcomes for leiomyosarcoma patients continue to be disappointing, and survival prediction results and related predictive factors based on small sample reports have been inconsistent^23–26^. RLS patients have a low 5-year survival rate and a poor prognosis, primarily due to the specific anatomical location of the retroperitoneum. The retroperitoneal space extends from the diaphragm to the extra-peritoneal space in the pelvis. Tumours tend to grow insidiously, with inconspicuous clinical manifestations, resulting in the quick formation of giant tumours that are difficult to remove in their entirety, and are prone to recurrence and distant metastasis, making diagnosis and treatment difficult. Therefore, an accurate survival rate prediction for RLS patients is critical for effective clinical management and medical decision-making.

In practice, it is difficult to predict a patient's OS using a single prognostic factor; however, a combination of several independent prognostic factors can improve the prediction accuracy. Traditional staging systems, such as the AJCC TNM staging divide patients into four stages based on the all-or-nothing principle of classifying variables. They are, however, no longer the most effective way of categorising RLS patients into different prognosis groups^27^. Hence, a new predictive model is required. The nomogram, which uses a simple graphical representation to provide an individualised prediction of survival results, has become an essential part of modern medical decision-making^28,29^. The nomogram incorporates multiple prognostic variables, including continuous variables, and depicts the factors and outcomes associated with more complex relationships between prognoses^30^. Furthermore, compared to the SEER stage, the nomogram has a more robust predictive ability. Previous research has used nomograms for retroperitoneal sarcoma and leiomyosarcoma in non-retroperitoneum parts to guide patient management^1,12,31^. Patients with RLS, on the other hand, do not have a nomogram to follow. The poor prognosis and uniqueness of disease location of RLS patients necessitate the creation of a nomogram for estimating their OS.

In this study, 305 RLS patients were recruited from SEER database. A nomogram was developed to predict RLS patients' OS at 1, 3, and 5 years. The nomogram showed good discrimination in both the training and validation sets, and the predicted survival rate aligned with the actual survival rate. The DCA analysis also revealed that nomogram had high clinical utility as a practical predictive tool. Furthermore, the developed risk stratification system supplemented the nomogram, allowing researchers to identify RLS patients at high mortality risk and provide early warning for better management. RLS is difficult to eradicate, so recurrence and distant metastasis complicate the diagnosis and treatment. As a result, an accurate survival rate prediction for RLS patients appears to be essential for effective clinical management and medical decision-making.

In the univariable Cox regression analysis, surgery, AJCC T stage, radiotherapy, chemotherapy, tumour size, tumour stage, and tumour grade were related to the OS of RLS patients (*p* < 0.05). In addition, surgery, tumour size, tumour grade, and tumour stage were identified as independent prognostic factors of RLS in the multivariable Cox regression analysis. Even though the exact pathogenesis of RLS is unknown, the only curative modality for treating RLS is surgery, which includes complete excision of the primary tumour and reduction of local recurrence^4^. Recent surgical advancements increased the 5-year survival rate of retroperitoneal sarcoma from 47.0% in 1998–2005 to 58.4% in 2002–2012 and the 10-year survival rate from 27.0 to 45.3%^32,33^. However, most RLS patients presented giant tumours at diagnosis, associated with a poor prognosis. According to An et al*.*, patients with retroperitoneal tumours less than 10 cm had a 78% 5-year survival rate, whereas those with tumours larger than 10 cm had a 38% 5-year survival rate^34^. Furthermore, tumours easily adhere to the surrounding blood vessels and organs, making it difficult to separate them from the adjacent tissues, and intra-abdominal or distant metastasis can occur due to the complex structure of the retroperitoneal space. In particular, tumors adjacent to the inferior vena cava frequently result in tumours that cannot be resected at R0. Complete resection, namely, R0 and R1 resection, is the currently accepted surgical resection goal^35^. Although studies have not proven a survival difference between R0 and R1 resection, R2 resection has a poor prognosis^35^. Moreover, systemic metastasis is the most common cause of postresection failure in RLS. However, the OS of RLS patients with distant metastasis is significantly longer than patients with other sarcomas. As a result, surgical intervention for distant metastasis is still needed to reduce complications and improve patient quality of life, especially for those with low- and middle-grade tumours^35,36^. Patients with high-grade tumours with early intra-abdominal recurrence, more frequent systemic metastases, and aggressive biological characteristics, may not benefit from extensive surgery. They may require other adjuvants or systemic treatment strategies.

The role of adjuvant radiotherapy and chemotherapy in RLS treatment is still under investigation, but its unified recommendation is still up for debate. Compared to surgery alone, proponents prefer adjuvant radiotherapy and chemotherapy to improve local symptom control^37,38^. Concurrently, opponents challenge the benefits and claim that they did not increase the OS rate but instead brought side effects^13,39–41^. There is currently no level 1 evidence to support adjuvant radiotherapy and chemotherapy for the treatment of primary RLS. Therefore, it is also difficult to extrapolate the absolute benefits of radiotherapy and chemotherapy from historical data because various factors influence treatment outcomes. The proper selection of patients for perioperative radiotherapy and chemotherapy is still being researched. Meanwhile, multi-center randomised trials are urgently needed to determine the best clinical strategy for improving local control in RLS patients. High-grade tumours are a risk factor for systemic metastasis and poor prognosis in RLS patients. According to Lewis et al*.*, the median OS of patients with high-grade retroperitoneal sarcomas was 33 months, while patients with low-grade were 149 months^9^. Our research findings also revealed a better prognosis for patients with low-grade tumours than those with high-grade tumours. Furthermore, patients with distant metastases have a lower survival rate than patients with local or regional metastases. This trend illustrates the significance of the early diagnosis of RLS patients.

Despite the excellent forecasting performance of the nomogram and risk stratification system, this study has unavoidable limitations. First, it is based on retrospective research data, which may have a selection bias. Secondly, some information is missing in the SEER database, such as comorbidities, the severity of other organ metastases, surgical margin status, vascular invasion, and postoperative treatment. Finally, there are no other independent large-scale data sets to verify the nomogram and increase its applicability externally. In the future, it will be necessary to continuously enrich the information in the SEER database, increase the external verification of larger sample data sets, and constantly modify and improve the predictive value of the nomogram model.

## Conclusion

In summary, a nomogram model and a risk stratification system for predicting the 1, 3, and 5 years OS of RLS patients were developed using a large population-based cohort. The nomogram model demonstrated high prediction accuracy and significant clinical utility, suggesting that it could be used as an OS prediction tool and serve as a valuable reference for clinicians to formulate treatment plans for patients with RLS.

## Supplementary Information

Supplementary Figures.

Supplementary Table S1.

## Data Availability

The dataset from the SEER database that was generated and/or analyzed during the current study is available in the SEER dataset repository (https://seer.cancer.gov/). The datasets generated during and/or analyzed during the current study are available from the corresponding author on reasonable request.

## Abbreviations

SEER: Surveillance, epidemiology and end results
RLS: Retroperitoneal leiomyosarcoma
OS: Overall survival
ROC: Receiver operating characteristic
DCA: Decision curve analysis
AUC: Area under the curve
AJCC: American Joint Committee on Cancer
HR: Hazard ratio
CI: Confidence interval
TNM: Tumor-node-metastasis
