# Supplementary material for: A novel nomogram model to predict the overall survival of patients with retroperitoneal leiomyosarcoma: a large cohort retrospective study
Source: Sci Rep. 2022 Jul 13;12:11851. doi: 10.1038/s41598-022-16055-z (PMC9279432; doi:10.1038/s41598-022-16055-z)
Supplement: Supplementary file 1 — Supplementary Figures. [file 41598_2022_16055_MOESM1_ESM.docx]

**Supplementary Figures**


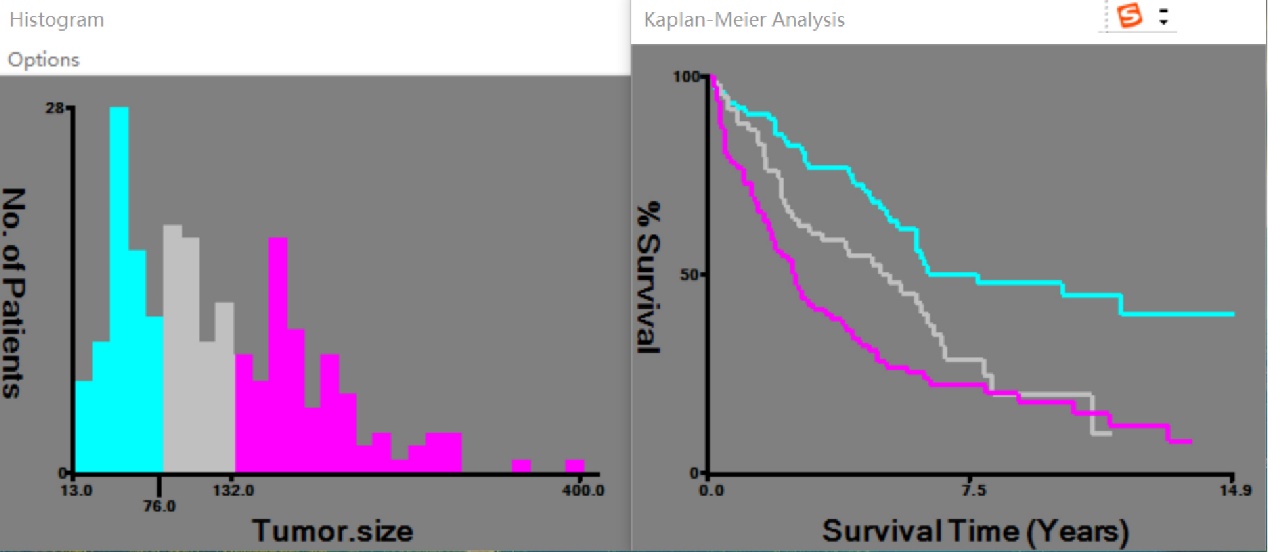


**Supplementary figure 1:** According to the X-tile software, the best cut-off values for the tumor size were determined to be 76 and 132 (mm).


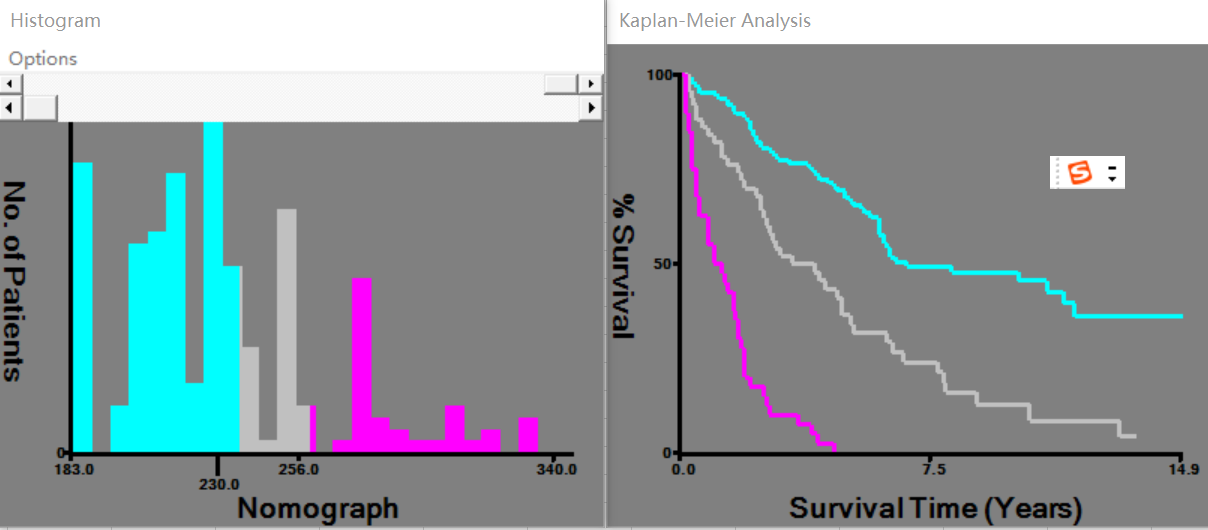


**Supplementary figure 2:** According to the X-tile software, the best cut-off values for the overall survival point were determined to be 230 and 256.
