# Supplementary material for: A novel nomogram model to predict the overall survival of patients with retroperitoneal leiomyosarcoma: a large cohort retrospective study
Source: Sci Rep. 2022 Jul 13;12:11851. doi: 10.1038/s41598-022-16055-z (PMC9279432; doi:10.1038/s41598-022-16055-z)
Supplement: Supplementary file 2 — Supplementary Table S1. [file 41598_2022_16055_MOESM2_ESM.docx]

**Table S1.** The detailed point of each independent prognostic factor in the nomogram.

| **OS-related independent variables** | **Corresponding point assignments in OS nomogram** |
| --- | --- |
| **Tumor size (mm)** | |
| <76 | 61 |
| 76-132 | 87 |
| >132 | 95 |
| **Tumor grade** | |
| Grade Ⅰ/Ⅱ | 61 |
| Grade Ⅲ/Ⅳ | 81 |
| **Tumor stage** | |
| Localized | 61 |
| Regional | 74 |
| Distant | 100 |
| **Surgery** | |
| No | 61 |
| Yes | 0 |

OS: overall survival
